# Transport Mechanisms at the Blood–Brain Barrier and in Cellular Compartments of the Neurovascular Unit: Focus on CNS Delivery of Small Molecule Drugs

**DOI:** 10.3390/pharmaceutics14071501

**Published:** 2022-07-20

**Authors:** Patrick T. Ronaldson, Thomas P. Davis

**Affiliations:** Department of Pharmacology, College of Medicine, University of Arizona, Tucson, AZ 85724-5050, USA; davistp@arizona.edu

**Keywords:** ATP-binding cassette transporters, blood–brain barrier, drug delivery, ischemic stroke, SLC transporters

## Abstract

Ischemic stroke is a primary origin of morbidity and mortality in the United States and around the world. Indeed, several research projects have attempted to discover new drugs or repurpose existing therapeutics to advance stroke pharmacotherapy. Many of these preclinical stroke studies have reported positive results for neuroprotective agents; however, only one compound (3K3A-activated protein C (3K3A-APC)) has advanced to Phase III clinical trial evaluation. One reason for these many failures is the lack of consideration of transport mechanisms at the blood–brain barrier (BBB) and neurovascular unit (NVU). These endogenous transport processes function as a “gateway” that is a primary determinant of efficacious brain concentrations for centrally acting drugs. Despite the knowledge that some neuroprotective agents (i.e., statins and memantine) are substrates for these endogenous BBB transporters, preclinical stroke studies have largely ignored the role of transporters in CNS drug disposition. Here, we review the current knowledge on specific BBB transporters that either limit drug uptake into the brain (i.e., ATP-binding cassette (ABC) transporters) or can be targeted for optimized drug delivery (i.e., solute carrier (SLC) transporters). Additionally, we highlight the current knowledge on transporter expression in astrocytes, microglia, pericytes, and neurons with an emphasis on transport mechanisms in these cell types that can influence drug distribution within the brain.

## 1. Introduction

A primary goal of neuropharmacology is optimal delivery of effective free drug concentrations to specific molecular targets in the brain. Over the past several years, researchers have attempted to exploit the physiology of the blood–brain barrier (BBB) to achieve this objective. For large molecule therapeutics such as proteins, targeting receptor-mediated transcytosis mechanisms (i.e., transferrin receptors (TfRs) and insulin receptors) has resulted in many successful preclinical studies [1,2,3,4,5]. For example, Chang and colleagues demonstrated that an erythropoietin-TfR antibody fusion protein (cTfRMAb-EPO) could cross the BBB, decrease amyloid peptide load in the hippocampus, and improve spatial memory in male APP/PS1 mice aged 5.5 months [3]. More recently, a BBB transport vehicle engineered to bind to the TfR apical domain was shown to successfully deliver an anti-β-secretase cargo peptide to the brain and produce a sustained pharmacodynamic effect both in human TfR-expressing C57BL6 mice and in cynomolgus monkeys [5]. While research on the blood-to-brain transport of large molecules is rapidly progressing, there have been few paradigm-shifting studies that have presented new delivery approaches for small molecules. In fact, such strategies for small molecule therapeutics remain focused on optimization of passive transcellular diffusion. This is often reflected by the continued application of Lipinski’s “Rule of 5”, which relates the BBB passive permeability to molecular weight, lipophilicity, polar surface area, hydrogen bonding capacity, and charge [6]. Using rational medicinal chemistry approaches, the “Rule of 5” guides drug design toward molecules that are less than 500 Da, possess clogP values between 1.5 and 2.5, have polar surface areas with an upper limit of 90 Å^2^, have fewer than five hydrogen bond donors and ten hydrogen bond acceptors, and are primarily uncharged at physiological pH [6]. Indeed, many efficacious CNS small molecule drugs possess these physicochemical properties [7,8]; however, passive diffusion is a non-selective process that does not provide the capability of precisely controlling drug delivery into brain tissue. This suggests that many detailed studies are required to identify transport mechanisms that can enable selective drug uptake across the BBB. Success in this area will lead to safer and more effective pharmacological treatments for neurological diseases.

Over the past several years, endogenous BBB transporters have been evaluated due to their importance as determinants of CNS drug disposition and drug efficacy. Most of the published research on BBB transporters has focused on those proteins that restrict therapeutic uptake into brain tissue (i.e., efflux transporters). The panel of efflux transporters that are known to be functionally expressed in brain microvascular endothelial cells include members of the ATP-binding cassette (ABC) superfamily, such as P-glycoprotein (P-gp), breast cancer resistance protein (BCRP in humans; Bcrp in rodents), and multidrug resistance proteins (MRPs in humans; Mrps in rodents) [9,10]. This is primarily due to the concept that efflux transporter liability, particularly with respect to P-gp, is a discriminating factor between centrally active drugs and non-CNS drugs [7]. Hence, it was hypothesized that blockade of BBB efflux transporters could result in improvement in brain penetration for drugs, particularly those with low passive permeability and moderate-to-high P-gp transport liability. Problematically, clinical trials targeting P-gp with pharmacological inhibitors (i.e., verapamil, cyclosporine A, and valspodar) have failed due to inhibitor toxicity and/or the enhanced penetration of drugs into non-CNS tissues [9,11,12]. P-gp is ubiquitously expressed throughout the human body. Epithelial tissues that functionally express P-gp include the colon, small intestine, renal proximal tubules, and bile canaliculi. This is an important consideration because blocking P-gp at the BBB using pharmacological antagonists will also block its activity in these other tissues, resulting in increased systemic drug disposition and an enhanced probability of off-target effects and/or dose-limiting toxicities [12,13]. A more rational approach for effective CNS drug delivery involves targeting transport mechanisms with the directionality to move substrates out of the systemic circulation, across the endothelial cell, and into brain parenchyma. To achieve this goal, a detailed evaluation of solute carrier (SLC) transporters that include centrally acting drugs amongst their substrate profile (i.e., organic anion-transporting polypeptides (OATPs in humans; Oatps in rodents), organic cation transporters (OCTs in humans; Octs in rodents), and multidrug and toxin extrusion transporters (MATEs in humans; Mates in rodents)) is necessary. Understanding the dynamics of CNS drug delivery also requires an appreciation that both efflux and influx transporters are expressed in other brain cellular compartments (i.e., glial cells, pericytes, and neurons). Such transporters play a critical role in brain drug distribution and, therefore, can have profound effects on the treatment of neurological disorders. In this review, we summarize the current knowledge on the localization and functional expression of those ABC and SLC transporters relevant to CNS drug delivery and drug distribution. Our focus will include both the BBB and brain parenchyma to emphasize how the rigorous comprehension of transporter function in the brain can lead to the optimization of blood-to-brain drug transport and the improved treatment of neurological diseases. We will use the example of ischemic stroke to demonstrate this critical concept.

## 2. Overview of ATP-Binding Cassette (ABC) and Solute Carrier (SLC) Transporters at the Blood–Brain Barrier (BBB)

For many centrally acting drugs, selective uptake into the brain and efflux from the brain is mediated by transport proteins. To date, several transport systems have been identified at the BBB. These include members of the ABC and SLC superfamilies, which function to determine the types of molecules that can access the brain while simultaneously restricting the CNS accumulation of others. Below, we provide an overview of these transport systems and their relevance to CNS drug delivery.

### 2.1. ABC Transporters

**Figure 1:**
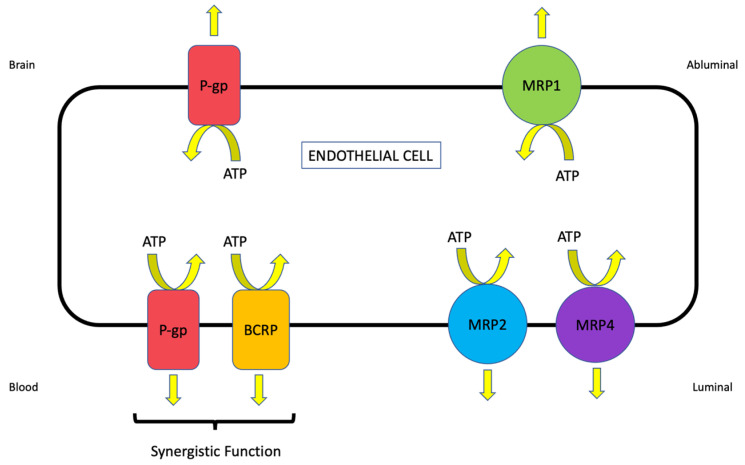
Localization of ABC Transporters in Brain Microvessel Endothelial Cells. Adapted with permission from Nilles et al. ref. [17]. 2022. Copyright 2022 by the authors.

The ABC transporter superfamily is among the largest and most ubiquitously expressed protein families that have been discovered. These transporters are grouped into seven distinct subfamilies (i.e., ABCA-ABCG) based upon the sequence homology of their nucleotide-binding domains and transmembrane domains, gene structure, and domain order [14]. ABC transporters are involved in various physiological processes, including lipid bilayer maintenance, peptide transport, and sterol transport. The most clinically relevant role of ABC transporters is their direct contribution to the development of the multidrug resistance (MDR) phenotype [15]. In the seminal work published by Michael Gottesman, the MDR phenotype was defined as the simultaneous resistance to several structurally unrelated compounds that do not result from independent genetic mutations that confer resistance to a single therapeutic agent [16]. Perhaps the most well-studied ABC transporter is P-gp, a principal contributor to the MDR phenotype and a clinically relevant impediment to drug permeation across the BBB. Other ABC transporters that are known to be involved in CNS drug disposition include BCRP/Bcrp (also known as ABCG2) and MRP/Mrp isoforms. The localization of critical ABC transporters that are expressed at the brain microvascular endothelium is depicted in Figure 1.

#### 2.1.1. P-glycoprotein (P-gp)

P-gp was first discovered by Dr. Victor Ling at the University of Toronto via the identification and characterization of a novel subclone of Chinese hamster ovary cells that displayed reduced cellular permeability and subsequent chemoresistance to the anti-gout drug colchicine [18]. Since this paradigm-shifting observation, P-gp has been shown to be a 170 kDa transmembrane glycoprotein that possesses two homologous halves, each with an intracellular ATP-binding site [19]. The efflux transport of drugs by P-gp requires ATP hydrolysis, which indicates that this protein functions as a primary active transporter. This transporter is encoded by the *MDR/mdr* gene, which has two isoforms in humans (i.e., *MDR1* and *MDR2*) and three isoforms in rodents (i.e., *mdr1a, mdr1b,* and *mdr2*). *MDR2* and *mdr2* are primarily expressed in the liver and are involved in transport of phosphatidylcholine into the bile. P-gp encoded by *MDR1*/*mdr1a*/*mdr1b* has evolved as a protective mechanism that prevents the CNS uptake of potentially toxic xenobiotics, a function that is highlighted by observations obtained using P-gp null (i.e., *mdr1a/mdr1b* (−/−)) mice. In rat and human brain tissue, P-gp has been shown to be localized at both the luminal and abluminal plasma membrane of microvascular endothelial cells [20]. While P-gp functional expression at the luminal plasma membrane is well-established to restrict xenobiotic uptake into brain tissue, its role at the abluminal membrane is less clear. Interestingly, immunogold cytochemistry revealed that the density of P-gp antigenic sites at the abluminal plasma membrane was 1.4-fold greater than at the luminal membrane [20]. This may reflect a role for P-gp in directing substances toward brain interstitial fluid to maintain their CNS concentrations. In the presence of ivermectin (i.e., a neurotoxic pesticide), P-gp null mice exhibit a 100-fold increase in brain accumulation as compared to wild-type controls [21]. Furthermore, P-gp null mice treated with ivermectin present with neurological symptoms that reflect central toxicity, including tremors, paralysis, coma, and death [21]. More recently, positron emission tomography (PET) technology has enabled the real-time study of P-gp function in experimental animals. For example, administration of the competitive P-gp inhibitor tariquidar resulted in increased CNS distribution of [^11^C]metoclopramide and [^11^C]verapamil by 2.1-fold and 2.4-fold, respectively [22]. P-gp expression has been demonstrated in cultured human vascular endothelial cells [23,24,25], in human brain cortical microvessels [26,27,28,29], and at the BBB in human cerebral tissue fixed in situ [20]. The advent of PET technology for the study of drug transport mechanisms has also enabled the evaluation of P-gp activity in living human subjects. For example, tariquidar was shown to increase the distribution volume and influx rate constants for [^11^C]verapamil in five healthy human volunteers [30]. An important caveat to this study was that P-gp inhibition at the BBB was reported to be “far from complete” as compared to peripheral P-gp inhibition [30], an observation that adds to the body of literature describing challenges associated with the blocking P-gp function for the enhancement of CNS drug uptake. Similar results were obtained by Muzi and colleagues, where increased cerebral blood flow-corrected brain uptake of [^11^C]verapamil was demonstrated in the presence of the P-gp inhibitory drug cyclosporine A [31]. Therapeutic concentrations of quinidine, a competitive P-gp inhibitor, were also shown to increase CNS delivery of [^11^C]verapamil by PET imaging; however, the P-gp inducer rifampin did not affect P-gp activity at clinically relevant concentrations [32]. These observations should not be interpreted to imply that induction of P-gp transport activity does not occur at the human BBB. Rather, these data point toward a need to test other drugs that have been shown to modulate P-gp transport activity in preclinical studies so that a detailed understanding of this critical transporter can be obtained.

P-gp has a vast substrate profile that renders it a formidable obstacle to CNS drug delivery. In fact, the number of compounds known to be P-gp substrates is continuously expanding in direct correlation with the advancement of the BBB field. Most P-gp transport substrates are non-polar, weakly amphipathic compounds. Indeed, P-gp can recognize and transport drugs with varying structures and sizes that range from 250 to 1202 Da [9,33]. The list of known substrate categories includes, but is not limited to, antibiotics, calcium channel blockers, cardiac glycosides, chemotherapeutics, immunosuppressants, anti-epileptics, anti-depressants, opioid analgesic drugs, statins (i.e., HMG CoA reductase inhibitors), and HIV-1 protease inhibitors [9,10,15]. For example, uptake of atorvastatin and rosuvastatin by human umbilical vein endothelial cells (HUVECs) was shown to be increased in the presence of pharmacological P-gp inhibitors (i.e., cyclosporine A, PSC833), a “mixed” P-gp/Bcrp inhibitor (i.e., GF120918), and metabolic inhibitors (i.e., sodium azide and dinitrophenol), suggesting that these two statins are substrates for P-gp [25]. Additionally, P-gp has been shown to be a critical determinant of brain penetration for morphine [34] and experimental opioid analgesic peptides such as D-Pen^2^,D-Pen^5^]enkephalin (DPDPE) [35]. The brain distribution of the currently marketed catechol-O-methyltransferase (COMT) inhibitor tolcapone and a novel COMT inhibitor (i.e., BIA 9-1079) were demonstrated to be increased in the presence of the P-gp inhibitor drug elacridar [36], suggesting that P-gp is also an important determinant of blood-to-brain transport for this class of centrally acting drugs.

#### 2.1.2. Breast Cancer Resistance Protein (BCRP/Bcrp)

BCRP was originally identified in the MCF-7/AdrVp breast cancer cell line that was generated to study novel pharmacological approaches to circumvent the MDR phenotype [37]. Despite the lack of expression of P-gp or MRP1 in these cells, ATP-dependent efflux transport of adriamycin and rhodamine 123 was observed, indicating the presence of a novel transporter protein [38,39]. This novel transporter was later cloned from MCF-7/AdrVp cells and termed “breast cancer resistance protein [40]”. Bcrp is comprised of 655 amino acids and has a molecular weight of approximately 72 kDa. As such, it is often referred to as a “half-transporter” that must form homo- or heterodimers to efflux drugs [41,42]. At the BBB, BCRP/Bcrp has been shown to be expressed at the luminal plasma membrane of brain microvascular endothelial cells [43,44,45]. Using absolute quantitative targeted proteomics, BCRP was detected at quantifiable levels in a human brain microvessel endothelial cell line (hCMEC/d3) [46] and in human brain microvessels [47]. The substrate profile of BCRP/Bcrp often overlaps with that of P-gp, thereby enabling these two transporters to function in synergy to limit the CNS uptake of selected drugs [25,36,48,49,50,51]. For example, rosuvastatin is a transport substrate for both P-gp and Bcrp, a fact that greatly affects its CNS distribution [25,49]. Using triple knockout mice (i.e., *mdr1a/b(−/−); Bcrp (−/−)*), Laramy and colleagues demonstrated that the total and free brain-to-plasma concentration ratios for the anti-cancer drug ponatinib were 15 times higher in the knockouts as compared to the wild-type controls, an observation that experimentally emphasizes the synergistic interplay between these two critical efflux transporters [51]. Similar results have been reported in the literature for vemurafenib [52] and sorafenib [53]. Additionally, the HIV-1 protease integrase inhibitor raltegravir was reported to be transported by both P-gp and BCRP in the hCMEC/d3 cell line, suggesting a potential synergistic effect of these ABC transporters [54]. In contrast, CNS uptake of many opioid analgesic drugs is limited by P-gp but not Bcrp [10]. Therefore, it is imperative that these critical efflux transporters be studied together to determine whether synergistic efflux transport involving P-gp and Bcrp exists for individual compounds and how such an effect can modulate CNS drug disposition and/or efficacy. In addition to therapeutic agents, the substrate profile for Bcrp includes endogenous and/or natural product substances such as steroids [55], glutathione [56], folate [57], and phenethyl isothiocyanate (PEITC) [58].

#### 2.1.3. Multidrug Resistance Proteins (MRPs/Mrps)

The primary role of Mrps is to extrude substances from cells, thereby providing additional transporter contributors to the development of the MDR phenotype. MRPs/Mrps differ from P-gp in that their substrate profile is restricted to organic anions and their glucuronidated, sulfated, and glutathione-conjugated metabolites [15]. Delineating specific properties and functional significance of individual MRP/Mrp isoforms is challenging due to the existence of multiple homologues with overlapping substrate profiles. At the BBB, the functional expression of Mrp1, 2, 4, and 5 has been confirmed and Mrp3 and 6 may also contribute to brain-to-blood drug transport at the microvascular endothelium [59]. The presence of MRP/Mrp homologues at the luminal side of the BBB is likely a critical determinant in controlling the delivery of drugs to the brain. For example, Mrp downregulation at the BBB was shown to result in the enhanced analgesic potency of morphine following systemic administration [60]. Using Mrp4(−/−) mice, Kanamitsu and colleagues demonstrated that this ATP-dependent efflux transporter was a critical determinant of brain penetration for therapeutic agents such as ochratoxin A, pitavastatin, raltitrexed, pravastatin, and cyclophosphamide [61]. Additionally, the ability of Mrp1, Mrp2, and Mrp4 to actively efflux the endogenous antioxidant glutathione may have implications in neurological diseases that feature an oxidative stress component. Glutathione is responsible for maintenance of cellular redox balance and antioxidant defense in the brain. It has been previously shown that Mrp functional expression is upregulated in response to oxidative stress conditions, an effect that promotes cellular glutathione extrusion [62]. The enhanced functional expression of Mrp isoforms at the BBB can cause reduced brain and/or endothelial cell concentrations of glutathione, altered cellular redox status, and increased potential for cellular injury and death. Prostaglandin D_2_ (PGD2), an eicosanoid mediator involved in the exacerbation and progression of neuroinflammation, has also been identified as an endogenous transport substrate for Mrp4 [63]. This was demonstrated by the observation that Mrp4 inhibitors (i.e., probenecid, benzylpenicillin, and cefmetazole) reduced the brain efflux index (BEI) of [^3^H]PGD2 following intracerebral injection in male Wistar rats [63].

### 2.2. Solute Carrier (SLC) Transporters

**Figure 2:**
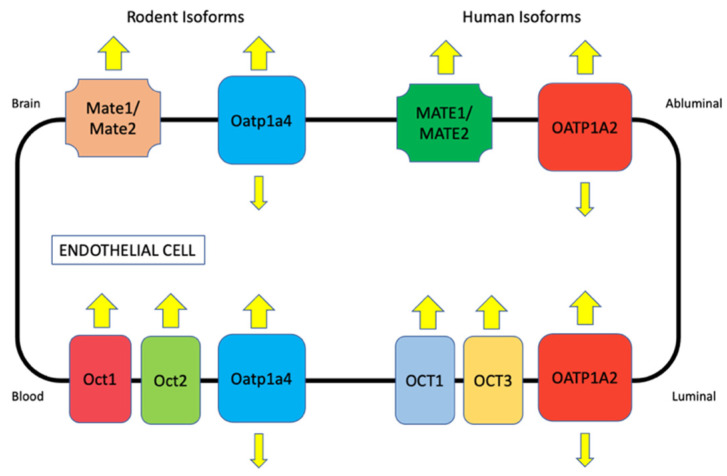
Localization of SLC Transporters in Brain Microvessel Endothelial Cells. Adapted with permission from Nilles et al. ref. [17]. Copyright 2022 by the authors.

A previous publication by the International Transporter Consortium provided a comprehensive analysis of the literature and suggested that effective clinical modulation of BBB efflux transporters using competitive inhibitors is highly unlikely [64]. This article came to this conclusion following a detailed review and analysis of multiple published preclinical studies and predicted that the maximal enhancement in drug uptake possible by blocking P-gp and BCRP will not exceed 2-fold [64]. This determination was based on the assumption that 100% of P-gp and BCRP transporters at the brain microvascular endothelium would need to be inhibited, an impossible goal given the highest clinical doses of efflux transporter inhibitors that could ever be administered to a patient [64]. This hypothesis has profound implications for CNS drug delivery as it suggests that blocking transporters that move substrates out of the brain is not a viable translational strategy to optimize the quantity of drug that gets into the brain. Inhibitor toxicity and increased drug penetration into systemic organs and tissues are additional obstacles that have prevented the advent of efflux transporter inhibition in the clinic. Therefore, our laboratory has proposed that a more rational strategy is to target those transporters that function to move their substrates in the blood-to-brain direction (i.e., uptake transporters). Indeed, selective CNS accumulation of many circulating substances requires SLC transporters. More than 400 SLC transporters that have been shown to be important for functions such as ion and nutrient uptake into the cell and drug absorption, distribution, metabolism, and excretion (ADME) have been identified in human cells/tissues [65,66]. Transport mechanisms for SLC transporters include facilitated diffusion (i.e., transport in the direction of substrate electrochemical gradient) or secondary/tertiary active transport (i.e., substrate transport that is dependent upon ion/solute gradients established by primary or secondary active transporters) [67]. SLC family members exhibit different specificities and affinities for transport substrates [68,69]. In this section, we will focus on OATPs/Oatps and OCTs/Octs, two types of SLC transporters that can be effectively targeted for drug delivery to the brain. The localization of critical OATP/Oatp and OCT/Oct transporters that are expressed in brain microvessel endothelial cells are presented in Figure 2.

#### 2.2.1. Organic Anion-Transporting Polypeptides (Oatps)

Due to their capabilities for efficient blood-to-brain delivery of small molecule drugs, our laboratory has extensively studied the protein expression and transport activity of OATPs/Oatps at the BBB. We have focused much of our work on Oatp1a4, the primary Oatp isoform responsible for drug transport at the BBB in rats [35,68,70,71,72,73]. Immunohistochemistry, confocal microscopy, and Western blot analysis have confirmed the localization and/or protein expression of Oatp1a4 at the cerebral microvasculature [35,74,75,76]. Of note, double-labeling immunohistochemistry studies with antibodies targeted against Oatp1a4 and glial fibrillary acidic protein (GFAP), a protein marker of astrocytes, demonstrated Oatp1a4 localization at the plasma membrane in brain capillary endothelial cells [77]. OATP1A2 is the human orthologue of Oatp1a4 and its expression has been reported in human brain microvessels [78]. In fact, global proteomics analyses of brain tissue from healthy human subjects have suggested that OATP1A2 is one of the most abundant transporters at the BBB [27]. OATP1A2 and Oatp1a4 are believed to function as carrier-mediated transporters where the driving force for substrate transport across a biological membrane is dependent upon the transmembrane electrochemical gradient. This mechanism suggests OATPs/Oatps can function as both influx and efflux transporters where the directionality of transport is dependent, in part, upon whether the intracellular substrate concentration is greater or less than the extracellular substrate concentration. The process becomes readily apparent under conditions where the OATP/Oatp functional expression is increased, such as disease states (e.g., pain and/or cerebral hypoxia) [35,70] or following the activation of intracellular signaling networks that can modulate OATP/Oatp protein expression and transport activity [71]. Indeed, enhanced OATP/Oatp-mediated transport is reflected by an increase in the uptake transfer constant (K_in_) and, simultaneously, a small but statistically significant augmentation of the efflux rate coefficient (k_out_) [35,70,71]. Additionally, it is important to consider that OATP2B1 has been detected at the BBB [79,80,81] and could potentially contribute to CNS drug permeation. While OATP2B1 localization in cerebral microvasculature has not been confirmed, it is likely that this transporter is expressed at the abluminal membrane and contributes to the movement of solutes out of the endothelial cell and into the brain parenchyma [81]. Relevant Oatp transport substrates that exert pharmacological/physiological effects in the brain include statins (i.e., atorvastatin, pravastatin, and rosuvastatin), prostaglandin E_2_, dehydroepiandrosterone sulfate (DHEAS), and estradiol-17β-glucuronide [17,25,82,83].

#### 2.2.2. Organic Cation Transporters (Octs)

The BBB has evolved multiple transport systems that can be exploited for CNS delivery of drugs with different physicochemical properties. These include proteins that can facilitate the blood-to-brain uptake of cationic substances, such as the organic cation transporters (OCTs in humans, Octs in rodents). Previous studies in cultured brain endothelial cells or brain microvessels have reported the expression of Octs at the mRNA level [84,85]. Protein expression for OCT1/Oct1, OCT2/Oct2, and OCT3/Oct3 has been confirmed in immortalized brain endothelial cell lines derived from murine (bEND3) and human (hCMEC/d3) cerebral microvasculature [86]. These results are consistent with data reported by Lin and colleagues, which demonstrated the expression of Oct1 and Oct2 at the luminal plasma membrane in primary cultures of brain endothelial cells isolated from adult male Wistar rats [87]. Using immunolabeling with fluorescently tagged antibodies and imaging by confocal microscopy, we have shown that Oct1 is localized to freshly isolated brain microvessels prepared from young (i.e., 3 month old) female Sprague-Dawley rats [67,69]. More recently, global proteomic analysis demonstrated quantifiable protein amounts for OCT1 (0.54 ± 0.06–0.58 ± 0.11 pmol/mg total protein) and OCT3 (0.62 ± 0.08 pmol/mg total protein) in microvessels derived from healthy postmortem human brain tissue [27]. In contrast, quantitative targeted proteomics has not been able to detect abundant quantities for OCT1 or OCT2 at the BBB in hippocampal Brodmann Areas 17 and 39 [28]. When these proteomics studies are rigorously compared, the disagreements between data sets imply differences in the BBB localization/expression for OCT transporters across different brain regions. Therefore, it is essential that such results be confirmed by molecular analyses and functional studies.

The translocation of cationic solutes across polarized epithelial/endothelial cell layers involves a two-step process involving different transporters that work in a cooperative manner. A pertinent example is found in epithelial cells of the renal proximal tubule, where synergy between two transporters (i.e., OCT2 and MATE1) is required for the efficient secretion of cationic substances [88]. While OCT2 is involved in the initial uptake at the plasma membrane, MATE1 functions to ensure the cationic substrate efflux into urinary filtrate. At the BBB, it has been reported that OCTs/Octs are expressed at the luminal plasma membrane of microvascular endothelial cells, an observation that is consistent with their role as the first step in blood-to-brain transport of cationic substances [69]. The OCT1/Oct1- and OCT2/Oct2-mediated uptake of N-methyl-4-phenyl-1,2,3,6-tetrahydropyridine (MPTP) has been shown in brain endothelial cells, evidence for the functional expression of these transporters at the BBB [87]. The fact that MPTP can efficiently permeate the BBB by an OCT/Oct-dependent mechanism implies that MATEs may also be functionally expressed at the level of the microvascular endothelium. Mate1 and Mate2 protein expression was detected at the BBB in C57BL6/129 mice [86] and Mate1 mRNA and protein expression was detected in brain capillaries isolated from male ddY mice [89]. More recently, Mate1 gene expression was shown in brain capillaries from various mouse strains, including Swiss, FVB, and C57BL6/JRj [90]. With respect to human endothelial cells, studies in the hCMEC/d3 cell line resulted in detection of MATE1 and MATE2 protein expression [86]. Additionally, the Western blot analysis of human brain tissue demonstrated MATE1 and MATE2 expression in the frontal cortex, caudate nucleus, and putamen [86]. In contrast, Chaves and colleagues were unable to show MATE expression in microvasculature derived from human glioma tissue [90]. Indeed, pathophysiological factors associated with cancer progression and/or pharmacological treatment with antineoplastic drugs could explain the variability in results between the work of Chaves and colleagues and other studies examining MATE expression at the BBB. Nonetheless, these discordant observations in the BBB expression of MATE/Mate isoforms suggests that detailed experiments are necessary to clarify the role of OCTs/Octs and MATEs/Mates in CNS drug delivery. Overall, the Oct/Mate system provides an excellent opportunity to deliver cationic therapeutics to the brain. Examples of such drugs include memantine, amantadine, metformin, pramipexole, selegiline, varenicline, and amisulpride [17,86,88,91,92].

## 3. Case Study on Transport at the Blood–Brain Barrier (BBB)—Ischemic Stroke

As reported by the Global Burden of Disease Study in 2019, there are approximately 12.2 million cases of stroke worldwide, which have resulted in the death of 6.55 million individuals [93]. Approximately 87% of all strokes are ischemic [94] and are characterized by pathophysiology resulting from vascular occlusion and subsequent reduction in oxygen and glucose supply to the affected brain region. This process leads to necrotic cell death of neural tissue in the infarction core and substantial injury to tissue surrounding the core (i.e., the ischemic penumbra) [95,96]. The penumbra is a primary target for drug treatment due to slower cell degradation [67,97,98]. Current FDA-approved treatments for ischemic stroke are either pharmacological (i.e., recombinant tissue plasminogen activator (r-tPA; alteplase)) or mechanical/surgical (i.e., endovascular thrombectomy (EVT)). Pharmacotherapy with r-tPA has many limitations, including a short therapeutic window (i.e., 4.5 h) and/or an enhanced risk for intracerebral hemorrhage [97]. In fact, the National Institute of Neurological Disorders and Stroke (NINDS) of the National Institutes of Health (NIH) reported that the risk of clinically significant bleeding in patients that were dosed with r-tPA was 6.4% as compared to 0.6% for patients given placebo [99]. Risk factors for intracerebral hemorrhage following treatment with thrombolytic drugs include stroke severity, hyperglycemia, time from onset of stroke symptoms to treatment, elevated systolic blood pressure, thrombocytopenia, and advanced chronological age [100]. Mechanical EVT has been an important surgical advancement in the treatment of ischemic stroke by providing improved cerebral reperfusion to stroke patients [101,102]; however, the advent of EVT has not completely prevented post-stroke disability [102,103,104]. It is important to consider that EVT, as well as the administration of r-tPA, involves the restoration of blood flow to ischemic brain tissue (i.e., recanalization). Paradoxically, recanalization is known to be associated with the exacerbation of neuronal damage. CNS injury following recanalization ranges in severity from infarction progression to the development of vasogenic edema or fatal hemorrhaging, factors that are associated with ischemia/reperfusion (I/R) injury [98]. I/R injury is associated with increased BBB permeability (i.e., paracellular “leak”), the activation of pathological mechanisms associated with cell death (i.e., apoptosis, autophagy, and necrosis), enhanced neuroinflammation, and the production of elevated levels of reactive oxygen species (i.e., oxidative stress) [96,105,106,107,108].

The routine application of r-tPA and EVT for acute ischemic stroke treatment suggests that therapeutic approaches capable of protecting neuronal tissue against injury and/or promoting repair following I/R injury are urgently required. Preclinical studies have attempted to address this need through evaluation of currently marketed drugs and experimental therapeutics using in vivo stroke models. Many of these studies have reported positive results demarcated by neuroprotection, prevention of cerebral infarction expansion, and/or improved functional neurocognitive performance. In fact, approximately 95% of studies published in the scientific literature between 1990 and 2018 have reported such outcomes [102]; however, only a single investigational drug (i.e., 3K3A-activated protein C (3K3A-APC)) has advanced to a multi-center Phase III clinical trial [109]. There are several reasons for the poor translation from animal models to successful clinical trials, including (i) the paucity of well-designed experiments to measure cognitive and motor outcomes; (ii) the use of doses in animal studies that are not consistent with those that would be able to be administered to human subjects; and (iii) the use of healthy young animals that are not representative of the vast majority of ischemic stroke patients who tend to be older and frequently present with at least one comorbid condition (i.e., diabetes mellitus, obesity, tobacco smoking, hypertension, and atrial fibrillation) [102,110]. An additional consideration is that studies examining drug efficacy in experimental ischemic stroke have not evaluated the role of endogenous BBB transporters in determining the blood-to-brain uptake of therapeutic agents.

Cerebral ischemia has been previously shown to be associated with altered expression and activity of the critical BBB efflux transporter P-gp. Of particular significance, Spudich and colleagues demonstrated that P-gp protein expression is elevated at the BBB as early as 3 h following transient middle cerebral artery occlusion (MCAO) [111]. Additionally, immunoblot analyses showed increased P-gp protein expression in the cerebral microvasculature following MCAO (90 min) as well as in cultured rat brain endothelial cells subjected to hypoxia/aglycemia [112]. Altered functional expression of P-gp, as well as other BBB transporters that are determinants of CNS drug delivery, can have profound implications for brain disposition and therapeutic effectiveness of drugs designed to treat ischemic stroke. Indeed, several drugs with neuroprotective properties are established substrates of P-gp. For example, P-gp is involved in the brain uptake and distribution of tanshinone IIA, a natural product compound that has shown potential in preclinical studies to protect against I/R injury [113]. More recently, in vitro studies in human umbilical vein endothelial cells (HUVECs) have identified the commonly prescribed statin drugs atorvastatin and rosuvastatin as P-gp transport substrates [25]. In contrast to P-gp, little is known as to how I/R injury affects BCRP/Bcrp. A single study where bovine brain endothelial cells were co-cultured with rat astrocytes showed decreased *Abcg2* mRNA in response to oxygen/glucose deprivation (OGD) conditions [114]. Interestingly, this reduction in transporter gene expression occurred as early as 4 h post-OGD treatment, but the *ABCG2* transcript expression returned to control levels after 24 h reoxygenation [114]. Since BCRP functions in synergy with P-gp to restrict the BBB permeability of stroke therapeutics such as statins [49], it is critical that this finding be validated using in vivo models of experimental ischemic stroke.

**Figure 3:**
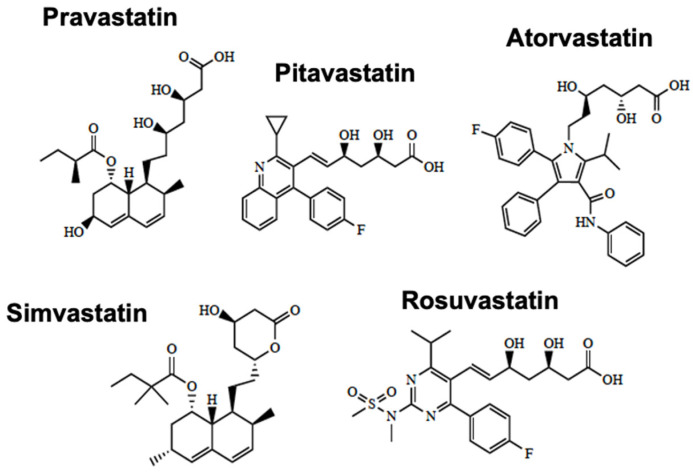
Chemical Structures of Currently Marketed Statin Drugs.

Despite the functional expression of efflux transporters at the BBB, SLC transporters represent a therapeutic opportunity to optimize the blood-to-brain uptake of drugs. Our laboratory has identified that OATPs/Oatps are critical transporters that enable the penetration of statins into the CNS. The chemical structures of currently marketed statins are shown in Figure 3. This mechanism is essential for these drugs to exert positive effects in the ischemic brain. Several in vivo studies performed by our group have provided evidence for BBB statin transport involving Oatps. For example, Thompson and colleagues reported that brain uptake of atorvastatin is mediated by Oatp1a4 in female Sprague-Dawley rats subjected to global hypoxia/reoxygenation stress (i.e., 6% O_2_ for 1 h followed by reoxygenation with 21% O_2_ for 10 min) [70]. Our results have demonstrated that both lipophilic statins (i.e., atorvastatin) and hydrophilic statins (i.e., pravastatin) can be transported by Oatp1a4 [71]. Interestingly, the Oatp-mediated transport of atorvastatin was at least 4-fold higher in female Sprague-Dawley rats as compared to their age-matched male counterparts [73], a study that provided the first evidence for sex differences of an endogenously expressed BBB transporter for drugs. These studies are congruent with previous observations from Oatp1a4(−/−) mice where the brain uptake of statin drugs (i.e., pitavastatin and rosuvastatin) was lower than that measured in the wild-type controls [115]. The evaluation of Oatp-mediated drug transport at the BBB in preclinical models requires the appreciation that species differences in drug transport properties can exist between rodents and human cells and/or tissues. An excellent example is the observation that OATP/Oatp transport properties for triptans, a class of medications designed to ease the symptoms of migraine or cluster headache, are dramatically unalike between humans and rodents [116]. Specifically, uptake transport for multiple triptan drugs (i.e., almotriptan, naratriptan, sumatriptan, rizatriptan, and zolmitriptan) was similar between Oatp1a4 null mice and wild-type controls, which implies that these drugs do not utilize an Oatp1a4-mediated transport mechanism to accumulate in the brain parenchyma [116]. In contrast, zolmitriptan was shown to be a substrate for OATP1A2, suggesting an OATP-mediated component of blood-to-brain uptake transport for this triptan drug [116]. This observation was confirmed in OATP1A2 knock-in mice where the CNS uptake of zolmitriptan was 1.6-fold higher as compared to age-matched wild-type controls [117]. In vitro studies in transfected Madin–Darby canine kidney (MDCK) II cells [116] and in HUVECs [25] showed that multiple statin drugs (i.e., atorvastatin, pravastatin, and rosuvastatin) are transport substrates for OATP1A2. These results emphasize the translational utility of using preclinical rodent models to study the transport of stroke drugs at the BBB if comparable properties between rodent transporters and their human orthologues have been demonstrated. Other OATP/Oatp transport substrates that may be effective as stroke therapeutics include opioid analgesic peptides. Using human-induced pluripotent stem cell-derived brain microvascular endothelial cells (iPSC-BMECs), Albekairi and colleagues showed that the cellular accumulation of the experimental opioid receptor peptide biphalin was decreased following exposure to estrone-3-sulfate, a known OATP inhibitor [118]. Both perinuclear and membranous expression of OATP1A2 was confirmed in iPSC-BMEC cells using fluorescent immunocytochemistry and flow cytometry [118]. Opioid receptor agonists such as biphalin have been demonstrated to exert neuroprotective effects in preclinical stroke models [119,120,121,122,123,124,125]. For example, Nozohouri and colleagues showed that biphalin can increase glutamate uptake in cultured mouse astrocytes and protect against BBB dysfunction in mice subjected to MCAO [125]. Therefore, targeting BBB OATP transporters represents a viable approach that can facilitate the delivery of potentially efficacious neuroprotective drugs to ischemic brain tissue.

**Figure 4:**
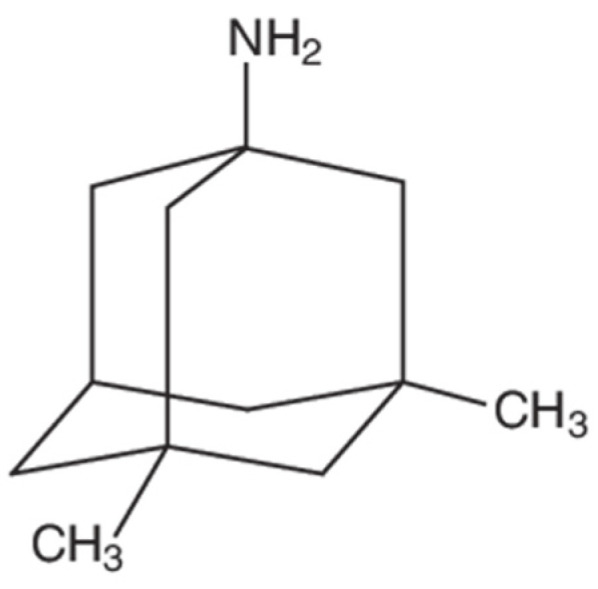
Chemical Structure of Memantine.

**Figure 5:**
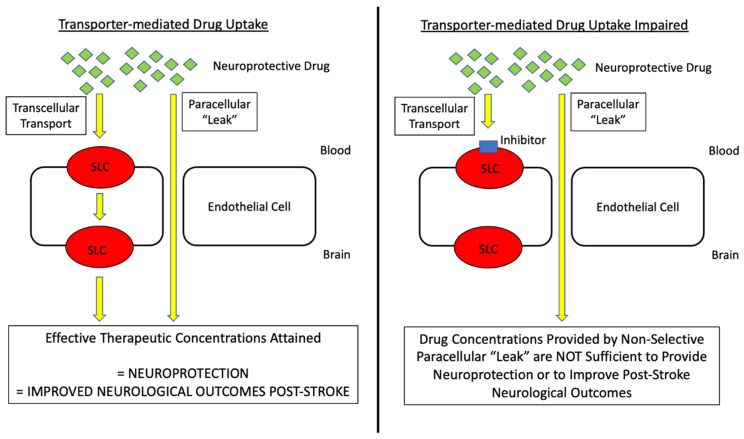
Interplay between SLC Transporter-Mediated Drug Uptake and Passive Paracellular Diffusion as Transport Mechanisms for Neuroprotective Drugs in Ischemic Stroke. Our data with memantine demonstrate that drugs that are transport substrates for SLC transporters such as Oct1/Oct2 require transporter-mediated blood-to-brain uptake to achieve efficacious concentrations following acute ischemic stroke. This leads to effective neuroprotection and an improvement in functional neurological performance during the acute phase of post-stroke recovery. When SLC transporter-mediated uptake is impaired, neuroprotective effects are attenuated despite the presence of “leak” into the brain via paracellular diffusion across the injured BBB.

The diversity of SLC transporters that are expressed at the BBB provides multiple opportunities to develop approaches for the improvement of CNS drug delivery. Memantine (Figure 4) is a known substrate for OCTs/Octs and, therefore, provides a useful tool to study the potential of targeting BBB transporters for cationic compounds. In terms of its mechanism of action, memantine functions as an antagonist of N-methyl-D-aspartate receptors (NMDARs). Reduced cerebral concentrations of oxygen and glucose following an ischemic insult can lead to an increased influx of calcium into neurons, a process that stimulates the release of the prototypical excitatory neurotransmitter glutamate. The excessive accumulation of glutamate within the synapse (i.e., excitotoxicity) is a primary cause of neuronal injury and/or death in the setting of ischemic stroke. The pharmacological inhibition of NMDARs protects against neuronal injury caused by excitotoxicity, which is the scientific premise for pharmaceutical development of memantine as a neuroprotective drug. At physiological pH, the majority of memantine molecules will carry a positive charge [92], a physicochemical property that indicates that a selective membrane transport mechanism is required for this small molecule drug to cross the BBB. Indeed, memantine has been shown to be transported by a specific system for cationic solutes in brain microvascular endothelial cells [92]. Studies in *Xenopus laevis* oocytes transfected with OCT2 mRNA demonstrated that memantine uptake was dependent upon a saturable transport mechanism (K_m_ = 34 ± 5 μM) [126]. Using memantine, we have shown for the first time that BBB transport is absolutely required for this drug to be efficacious in the setting of ischemic stroke [127]. Specifically, we report that memantine can improve functional neurological outcomes, reduce cerebral infarction progression, and decrease cerebral edema when administered as a single dose (5 mg/kg, i.v.) in male Sprague-Dawley rats subjected to MCAO [127]. Interestingly, the Oct transport inhibitor cimetidine attenuated all positive effects in our MCAO model [127], suggesting that Oct1/Oct2 transport is a primary mechanism for the neuroprotective effects of memantine. This is the first time that an endogenous BBB transport system has been shown to be required for a stroke drug to be effective, a concept that is depicted in Figure 5.

In the context of ischemic stroke, the role of transporters in cerebral microvasculature must be understood in conjunction with the knowledge that BBB dysfunction occurs 4–6 h following ischemic injury [96]. This pathological event is known to involve changes in localization and expression of transmembrane and intracellular proteins that comprise the tight junction. The modulation of tight junction complexes is manifested as the reorganization of oligomeric protein assemblies, which can lead to profound changes in BBB permeability and the exacerbation of ischemic injury to the brain [128,129,130]. For example, the decreased expression of claudin-3 and occludin (i.e., transmembrane proteins that are known components of tight junction protein complexes) was reported at the BBB in mice subjected to MCAO, an effect that led to the progression of a cortical infarction and the onset of cerebral edema [131]. The dysregulation of claudin-5 and ZO-1 via the siRNA knockdown of A-kinase anchor protein 12 (AKAP12) was shown to cause enhanced endothelial cell monolayer permeability to fluorescein isothiocyanate-labeled dextran (40 kDa) under in vitro OGD conditions [132]. Similar results were obtained in vivo where the conditional knockdown of NB-3 at the BBB resulted in the reduced protein expression of claudin-5, occludin, and ZO-1 and the subsequent enhancement in Evan’s blue-albumin extravasation [133]. Additionally, reduced dimerization and/or oligomerization of occludin was observed in rat brain microvessels in an in vivo model of hypoxia/reoxygenation stress, a component of stroke pathogenesis [129]. The result of the occludin movement away from the tight junction is increased paracellular diffusion (i.e., “leak”) of [^14^C]-sucrose, a vascular tracer with a molecular weight of 342 Da [129]. Under normal physiological conditions, tight junctions are responsible for the formation of a “physical barrier” between adjacent endothelial cells as evidenced by the restriction of paracellular diffusion to small molecule solutes as well as water [96,134,135]. This function requires the presence of transmembrane proteins (i.e., claudins, occludin) that can interact with each other to form a “seal” across the paracellular space that exists between apposing endothelial cells. Perhaps the most critical transmembrane tight junction protein is claudin-5 [130,136,137], whose essential role at the BBB is emphasized by the fact that claudin-5 knockout mice die within 10 h following birth [138]. The precise mechanism that causes death in these claudin-5 knockout mice is associated with the reduced control of a vascular “leak” in the cerebral microvasculature as evidenced by the increased extravasation of small molecule tracers (i.e., gadolinium and Hoechst 33258) that do not typically permeate the BBB [138]. In addition to claudin-5, brain microvascular integrity is maintained by transmembrane tight junction proteins such as occludin [128,129,139], tricellulin [140,141], and junctional adhesion molecules [142,143]. Intracellular accessory proteins such as ZO proteins, which are members of the membrane-associated guanylate kinase-like (MAGUK) protein family, interact with transmembrane tight junction proteins to facilitate linkage to the actin cytoskeleton. There are multiple publications that have shown the altered ZO-1 expression and/or localization and subsequent enhancement in BBB permeability following exposure to a pathophysiological stressor [144,145,146]. These observations imply that ZO-1 is essential to the maintenance of tight junction stability and function. 

Despite the knowledge that disruption of the tight junction can increase a non-selective paracellular “leak” at the BBB, proteins that facilitate the selective transport of small molecules can overcome these changes in microvascular integrity. This enables endogenous BBB transporters to remain as primary determinants of CNS drug disposition despite marked tight junction protein complex dysfunction. Relevant to ischemic stroke, our recent study with memantine shows that cimetidine can block its brain uptake in ipsilateral and contralateral cerebral cortices from both MCAO animals and Sham-operated controls; however, the magnitude of memantine uptake in the ipsilateral cortex was greater than that measured in the contralateral cortex under MCAO conditions [127]. This finding suggests that a component of blood-to-brain memantine uptake results from non-selective paracellular diffusion (i.e., “leak”). Indeed, we demonstrated increased brain uptake of sucrose, a small molecule vascular tracer that does not permeate the intact BBB [137], following MCAO in whole brain tissue and in the ipsilateral cerebral cortex [127]. It must be emphasized that cimetidine treatment blocked the ability of memantine to improve neurological performance and to limit the progression of cerebral infarction and brain edema [127]. Our laboratory has previously demonstrated this concept in another disease state (i.e., λ-carrageenan-induced inflammatory pain (CIP)). Using the CIP model [34], we have shown that tight junction protein complex integrity is disrupted and paracellular permeability to sucrose is enhanced following plantar injection of λ-carrageenan [139,147,148,149]; however, we also demonstrated that brain accumulation of morphine (i.e., a transport substrate for P-gp) in saline-treated animals and in λ-carrageenan-injected animals could only be increased in the presence of a competitive P-gp inhibitory drug (i.e., cyclosporine A) [34]. Taken together, these novel results imply that selective transport across microvascular endothelial cells (i.e., transcellular transport) remains a critical determinant of blood-to-brain drug delivery despite the opening of a non-selective paracellular diffusion route between adjacent BBB endothelial cells (Figure 5). In short, it can no longer be assumed that the BBB is “open” following a pathological insult such as ischemia/reperfusion injury.

## 4. Transport Mechanisms in Other Cell Types of the Neurovascular Unit

The BBB exists at the level of the brain microvascular endothelium. A central concept in BBB physiology is that endothelial cells cannot form barrier characteristics without coordinated communication networks with other CNS cell types [96,150,151]. Such networks gave rise to the concept of the “neurovascular unit (NVU)” that was formally defined by the National Institutes of Neurological Diseases and Stroke (NINDS) Stroke Progress Review Group in 2001 [152]. By emphasizing the co-operativity of cell–cell interactions between endothelial cells, glial cells (i.e., astrocytes, microglia), pericytes, and neurons as well as contributions from enzymes and proteins that comprise the extracellular matrix, the adoption of the NVU concept by the BBB field resulted in meaningful changes in the way that neurological diseases were studied [152]. It is now understood that glial cells and other CNS cellular constituents interact directly with brain microvascular endothelial cells to enable the development of a distinct barrier phenotype and to allow for rapid and dynamic responses to pathological and pharmacological stressors. This close relationship between neural and vascular function (i.e., neurovascular coupling) ensures that the brain receives an appropriate supply of oxygen and glucose and regulates other critical processes such as proteostasis, immune cell trafficking, temperature control, metabolic waste removal, and neurogenesis [153]. It is also important to note that transport proteins have been identified in these NVU cellular components. The isoforms of transporters in glial cells, pericytes, and neurons may differ from those expressed in endothelial cells; however, it is highly probable that these proteins work in concert with BBB transporters to control drug distribution within the CNS. Therefore, the functional expression of transporters in cellular compartments of the NVU requires more extensive evaluation in the setting of neurological diseases due to their potential role in modulating free drug concentrations at discrete molecular targets and, by extension, therapeutic effectiveness.

### 4.1. Astrocytes

Astrocytes are the most numerous CNS cell type and play an essential role in the development and maintenance of the BBB phenotype [154,155,156]. They are localized between neuronal cell bodies and endothelial cells and cover more than 99% of brain microvasculature with their end-feet [151]. The seminal work by Janzer and Raff in 1987 indicated that astrocytes may be the CNS cell type that is primarily responsible for preventing paracellular “leak” at the BBB [157]. Since this study was published, many others have demonstrated the central involvement of astrocytes in the regulation of the BBB. For example, the injection of a toxic chemical (i.e., 3-chloropropanediol) in male Fisher F344 rats resulted in focal astrocyte loss and a subsequent reduction in tight junction protein expression (i.e., claudin-5, occludin, ZO-1) and increased paracellular permeability to 10 kDa dextran [158]. In vitro, porcine brain endothelial cells showed improved BBB properties when co-cultured in the presence of an immortalized rat astrocyte cell line (CTX TNA2), suggesting that astrocytes produce and secrete trophic factors that stimulate the development of the BBB phenotype [155]. More recently, human iPSC-derived astrocytes were observed to reduce paracellular “leak” and improve the integrity of the tight junction protein complexes continuity when co-cultured with endothelial cells also generated from iPSCs [156]. More recently, imaging modalities such as two-photon microscopy have provided critical information on the anatomical relationship between astrocytes and microvascular endothelial cells at the NVU [159]. In addition to their role in the maintenance of the BBB phenotype, astrocytes are involved in the regulation of water and ion transport across the brain microvascular endothelium [160,161] and control of neurotransmitter (i.e., glutamate) concentrations in the synapse [151]. Additionally, astrocytes are known to express volume-regulated anion channels (VRACs). These channels release excitatory amino acids such as glutamate in a calcium-independent manner under pathological conditions that can lead to cellular swelling such as cerebral hypoxia [162] and ischemic stroke [163].

ABC transporters that are known to be expressed in astrocytes include P-gp [20,164,165], Bcrp [166], and MRP/Mrp isoforms [62,167,168]. Various MRP and OATP isoforms (i.e., OATP1A2, OATP1C1, OATP2B1, and OATP4A1) have been detected at the gene (i.e., mRNA) level in human glioma tissue [75]. Gao and colleagues observed that Oatp1a4 immunoreactivity did not colocalize with GFAP, suggesting that this transporter is not expressed in rat astrocytes [78]. In contrast, Oatp3a1 protein expression was detected in rat astrocytes, which implies a mechanism for prostaglandin and thyroxine transport in glial cells [169]. Several isoforms of organic cation transporters, including OCT3/Oct3, novel organic cation transporters (OCTNs in humans; Octns in rodents), and the plasma membrane monoamine transporter (PMAT in humans; Pmat in rodents) have been reported to be expressed in astrocytes [170]. The expression of these transporters in astrocytes suggests that these glial cells can play a critical role in the regulation of CNS drug distribution. That is, the complement of transporters in astrocytes may either sequester drugs within the cytoplasm (thereby preventing compounds from reaching their molecular target in the brain) or concentrate drugs in the brain extracellular fluid. The current knowledge in the field, as described by the glymphatic hypothesis, indicates that solutes are cleared from brain parenchyma by fluid flow mechanisms [171]. Supporters of the glymphatic hypothesis believe that this occurs via perivenous flow, a perception that is supported by the observation that tracer molecules entering the brain parenchyma via CSF or directly injected into the brain parenchyma will eventually distribute to the walls of large veins [171]. Opponents note that the fluid flow required to allow solutes to diffuse to the perivenous spaces is much greater than that supplied by CSF flow [171]. While we still do not have an adequate understanding of the mechanisms involved in the solute clearance from brain extracellular fluid, it is possible that passive diffusion and active transport processes are also involved. Additionally, it has been proposed that astrocyte cellular volume can fluctuate in response to physiological processes such as the sleep–wake transition, an effect that can lead to considerable changes in brain interstitial fluid volume [172]. Indeed, the release of fluid from astrocytes into the brain extracellular spaces can dilute drug concentrations and potentially affect the rate of transport by cellular compartments of brain parenchyma such as astrocytes. As such, the effect of astrocyte volume changes on drug transport and/or distribution in the brain requires further study.

### 4.2. Microglia

Microglia are the innate immune cells of the brain and comprise 10–15% of all cell types in the CNS [173]. Microglia belong to the myeloid lineage, which also includes monocytes/macrophages, neutrophils, and platelets [174]. The pathophysiological functions of microglia are best exemplified in the setting of cerebral ischemia where they participate in the initiation and maintenance of neuroinflammation but also post-stroke neural repair and inflammatory dampening [174,175,176]. In the context of normal physiology, microglia exist in a quiescent state and are not capable of endocytotic and/or phagocytotic functions. Morphologically, these resting microglia are ramified as demarcated by a small (5–10 μm) cell body and possess multiple thin radial processes [175]. The role of microglial processes under resting conditions are to sense the presence of pathological mediators and/or potentially toxic substances in the brain extracellular space [175]. The ability to employ state-of-the-art technologies such as two-photon microscopy imaging has enabled the visualization of radial processes while microglia are in a resting state [177]. Under pathophysiological conditions, microglia can quickly shift to an activated phenotype. The level of microglial activation is strongly correlated with the type and severity of the CNS disease state. Activated microglia are routinely described as existing in two functional states that are designated as M1-like and M2-like. M1-like microglia perform pro-inflammatory and pro-killing functions and are often perceived to be deleterious to neuronal integrity [176]. In contrast, M2-like microglia are involved in the regulation of the central immunity, inflammatory control, and repair/injury resolution [176]. It is important to point out that M2-like microglia are also capable of phagocytosis, which provides a unique ability for these cells to clear cellular debris in the brain and contribute to neural repair [178]. The M1/M2 classification system is imperfect and there are several exceptions as has been shown in several studies on neurological disease states where substantial heterogeneity in microglial phenotypes have been demonstrated [179,180]. Indeed, microglial activation is strongly correlated with BBB dysfunction, which is an early event in neurological diseases, including ischemic stroke [96], Alzheimer’s disease [181], and epilepsy [182].

Although previous studies have shown that microglia express ABC transporters, including P-gp, Mrp1, Mrp4, and Mrp5 [183,184,185], there are no published data outlining the expression of OATP/Oatp isoforms in microglia. In contrast, the uptake of ergothioneine was shown to be reduced in microglia isolated from Octn1 knockout mice, an observation that provides evidence for functional organic cation transporters in these glial cells [186]. Similar to astrocytes, the dynamic expression profile of transporters in microglia points toward a critical role in the regulation of drug distribution in the brain parenchyma. Clearly, more detailed studies on transporter functional expression in microglia are necessary to assess the ability of these cells to contribute to drug permeation and/or distribution in the CNS.

### 4.3. Pericytes

In addition to glial cells, pericytes play a fundamental role in the control of BBB homeostasis [187,188,189]. Pericytes are flat, contractile mural cells that are localized to the basement membrane of small blood vessels in the CNS [190,191]. Pericytes maintain direct contact with endothelial cells as demonstrated by pericyte ablation experiments, which result in morphological changes in adjacent pericytes [192]. Specifically, these cells will stretch to provide coverage to “open” areas of the microvascular endothelium where pericytes no longer exist [192]. Several contractile and cytoskeletal proteins, including α-smooth muscle actin (α-SMA), neural/glial antigen-2 (NG-2), vimentin, desmin, myosin, and nestin, are expressed by pericytes, observations that support the contractile nature of these cells [190,193]. It is important to point out that α-SMA content differs amongst pericytes, which suggests the existence of multiple pericyte populations (i.e., ensheathing pericytes, mesh pericytes, and thin-strand pericytes) which possess dissimilar characteristics [191]. Pericytes can be identified based on their expression of cell surface antigens, including the platelet-derived growth factor receptor-β, aminopeptidases N and alanyl aminopeptidase (CD13), regulator of G-protein signaling-5 (RGS5), and melanoma cell adhesion molecule (MCAM; also known as CD146) [190]. The role of pericytes in the maintenance of BBB properties is believed to involve the secretion of angiopoietin, which induces the expression of the critical tight junction protein occludin [194]. Using genetically modified mice that exhibit a decreased mural cell density, Armulik and colleagues showed that the magnitude of microvascular pericyte coverage is inversely proportional to the paracellular permeability at the BBB [195]. Specifically, increased endothelial pericyte coverage resulted in decreased extravasation (i.e., “leak”) of large molecular weight tracers, such as Evan’s blue-albumin, BSA-Alexa Fluor-555, IgG-DyLight 549, and horseradish peroxidase [195]. Enhanced BBB paracellular diffusion in response to pericyte loss differs across brain regions and the greatest permeability changes observed in the hippocampus, striatum, and cerebral cortex [196]. Although this study indicated that regional differences in the BBB leak did not result from dysfunction of tight junction protein complexes, a comprehensive examination of tight junction protein expression, localization, or dimerization/oligomerization in the pericyte-deficient *Pdgf-b^ret/ret^* mouse model was not conducted [196]. Furthermore, BBB permeability was only measured using the large molecule tracer IgG, and smaller molecular weight indicators of BBB permeability were not incorporated into the study design. Therefore, data presented in the study by Villasenor and colleagues are not sufficient to conclude that reduced pericyte coverage at the BBB did not cause tight junction dysfunction.

To date, data on the localization and functional expression of transporters in pericytes is limited. P-gp has been localized to the plasma membrane of pericytes in rat and human brain tissue fixed in situ [20]. It is also noteworthy that pericytes have been shown to induce the expression of P-gp in brain microvascular endothelial cells [189,197], further emphasizing their role in the maintenance of the BBB phenotype. The expression of Mrp1, Mrp4, and Mrp5 mRNA has also been reported in pericytes isolated from bovine brain tissue [198]. P-gp and MRP1 have also been detected at the protein level in postmortem brain tissue samples [199]. While the exact role of these transporters in pericytes has not yet been elucidated, it is highly possible that the functional expression of ABC transporters in this NVU cell type can contribute to the barrier properties of cerebral microvasculature by restricting the blood-to-brain uptake of drugs. Despite these intriguing findings with respect to ABC transporters, there are no published studies that describe the expression and/or function of OATPs/Oatps or OCTs/Octs in pericytes. This suggests a critical knowledge gap that must be addressed to fully understand the dynamics of the drug transport mechanism at the NVU.

### 4.4. Neurons

Neurons form the basic structural and functional component of the CNS. The primary function of neurons is to respond to stimuli by conducting electrical signals along conductive processes (i.e., axons). The conduction of electrical impulses results in the release of neurotransmitters that further regulate (positively and negatively) nearby neural responses [200]. This enables the brain to maintain a highly complex communication network. A few studies have reported expression of Oatp1a5 and Oatp2b1 in neurons isolated from rat and mouse brain [201]. The neuronal expression of Oatp2b1 is particularly compelling because many of its transport substrates (i.e., prostaglandins and leukotriene C_4_) are involved in inflammatory signaling and regulation [201]. Because neuroinflammation is a central component of multiple disease states, neuronal Oatp2b1 may represent a viable molecular target for the treatment of CNS diseases. Additionally, Oatp1a5 and/or Oatp2b1 may be involved in neurotoxicity due to their ability to transport cytotoxic microcystins [202]. Confocal microscopy imaging of human frontal cortical tissue demonstrated the expression of OATP3A1 in neurons at both the cell body and axon [169]. In vivo studies have shown that murine neurons express high levels of Oatp2a1 [203]. Additionally, OCT2 has been detected in human neurons [126], while Oct2 has been observed in murine neurons from the amygdala, dorsal raphe, frontal cortex, hippocampus, thalamus, median eminence, and pituitary gland [204,205]. In terms of efflux transporters, P-gp expression has been reported to be negligible in neurons but can be induced in response to pathological stressors [206,207,208]. In contrast, Mrp1 has been detected in primary cultures of murine prefrontal cortical neurons [209]. While these studies provide evidence for neuronal transporter expression, their role in CNS drug distribution and the effects on treatment effectiveness for neurological diseases requires more extensive evaluation. Such studies are warranted given the possible role of neuronal transporters in controlling drug distribution in the brain parenchyma and/or the access of therapeutic agents to their site of action.

## 5. Summary and Conclusions

The challenges of CNS drug delivery are best exemplified by ischemic stroke, which continues to be a significant cause of death and disability in the United States and worldwide. Since FDA-approved pharmacotherapy for ischemic stroke remains restricted to fibrinolytic therapy (i.e., r-tPA), safe and effective neuroprotective drugs are urgently needed. The application of these new chemical entities to the population of stroke patients is highly dependent upon transport across the BBB. Previous research by our laboratory has revealed several endogenous transporters with considerable potential to be targeted for optimization of CNS drug delivery; however, there are few studies in the preclinical stroke literature that describe the need to consider BBB transport mechanisms during therapeutic development. It is striking that statins and memantine, drugs that have shown varying degrees of success in stroke, are substrates for uptake transporters that are expressed at the human brain microvascular endothelium (i.e., OATP1A2 for statins; OCT1/OCT2 for memantine) while other compounds (i.e., disufenton sodium) that are not transported at the BBB have failed in the clinic [98]. Our laboratory has undertaken important studies to address this critical issue. In the case of memantine, we have been successful by demonstrating that membrane transport mediated by Oct1/Oct2 is a critical step that enables this drug to function as a neuroprotectant in the setting of ischemic stroke [127]. Our work has revealed that the BBB does not simply remain “open” and “leaky” following stroke. Rather, endogenous transporters are still capable of providing selective delivery of their solutes to ischemic brain tissue despite the existence of a route for paracellular diffusion. It is also important to consider that CNS disposition of a single drug is often determined by the combined activity of many different transporters that can move their substrates in opposite directions. Understanding the “multi-transporter environment”, both at the BBB and in cellular compartments of the NVU, is essential to moving the field forward by performing detailed and translational preclinical studies that can be developed into effective treatments for neurological diseases. This concept is emphasized by our recent publication where we rigorously evaluated the combined effects of Oatp1a4, P-gp, and Bcrp on the disposition of statin drugs (i.e., atorvastatin, pravastatin, and rosuvastatin) in the brain [49]. Taken together, these findings will facilitate the discovery of treatment strategies where small molecule neuroprotective drugs can be delivered safely and efficiently. Continued research in this area will enable preclinical stroke research to take a “giant step forward” via incorporation of drug transporter experiments into in vivo models of experimental stroke to understand how therapeutics can attain efficacious concentrations in the brain. The future discovery of new chemical entities and/or the development of neuroprotective treatment strategies for ischemic stroke will greatly depend upon obtaining a rigorous understanding of BBB transport mechanisms. Success in this area will also depend upon expansion of transporter studies to include other cell types of the NVU. Indeed, transport mechanisms in astrocytes, microglia, pericytes, and neurons can play a key role in determining drug distribution in the brain parenchyma and, by extension, the capability of a therapeutic agent to reach its site of action. Overall, endogenous transporters at the BBB/NVU represent an untapped opportunity that must be pursued to accelerate the development of pharmacological strategies for the treatment of neurological diseases such as ischemic stroke.

## Data Availability

Not applicable.
